# The Enigmatic Roles of PPR‐SMR Proteins in Plants

**DOI:** 10.1002/advs.201900361

**Published:** 2019-05-06

**Authors:** Yi Zhang, Congming Lu

**Affiliations:** ^1^ State Key Laboratory of Crop Biology College of Life Sciences Shandong Agricultural University Taian Shandong 271018 P. R. China

**Keywords:** pentatricopeptide repeat‐small MutSrelate (PPR‐SMR) proteins, RNA endonuclease, RNA manipulation

## Abstract

The pentatricopeptide repeat (PPR) protein family, with more than 400 members, is one of the largest and most diverse protein families in land plants. A small subset of PPR proteins contain a C‐terminal small MutS‐related (SMR) domain. Although there are relatively few PPR‐SMR proteins, they play essential roles in embryo development, chloroplast biogenesis and gene expression, and plastid‐to‐nucleus retrograde signaling. Here, recent advances in understanding the roles of PPR‐SMR proteins and the SMR domain based on a combination of genetic, biochemical, and physiological analyses are described. In addition, the potential of the PPR‐SMR protein SOT1 to serve as a tool for RNA manipulation is highlighted.

## 1 Introduction

Pentatricopeptide repeat (PPR) proteins are characterized by tandem helical repeats of a degenerate 31−36 amino acid motif, with 2−30 repeats per protein.^1^, ^2^ The PPR protein family was discovered through early, incomplete *Arabidopsis thaliana* genome analysis for proteins predicted to be targeted to chloroplasts and/or mitochondria.^3^, ^4^ Although further genome sequencing analyses revealed that PPR proteins are widespread in eukaryotes, PPR proteins are notably more abundant in plants than in other organisms.^4^, ^5^ More than 450 putative PPR proteins have been identified in *Arabidopsis thaliana* (Arabidopsis) and 477 in rice (*Oryza sativa*) whereas only six and five PPR proteins are predicted in human and yeast, respectively.^2^, ^5^, ^6^, ^7^ The reason for the striking expansion of the PPR family in plants remains unclear. The large number of PPR proteins in plants might be an evolutionary adaption for complex RNA metabolism within chloroplasts and mitochondria.^8^ Most PPR proteins bind RNA in a highly specific manner and thus play roles in post‐transcriptional control, such as RNA stabilization, RNA cleavage, RNA editing, RNA splicing, and translation in plant organelles.^7^, ^9^, ^10^

The PPR family is subdivided into two major subfamilies, P and PLS, based on motif structure. The P‐class subfamily members contain one or several canonical 35‐amino‐acid PPR (P) motifs. The PLS‐class subfamily members consist of characteristic triplets of P, L, and S motifs. The L and S motifs are related to the 35/36 amino‐acid and 31 amino‐acid PPR motifs, respectively.^2^, ^5^, ^11^ Occasionally, PLS‐class members contain additional, interspersed S motifs. Almost all PLS‐class members possess C‐terminal E domains following the last PPR motif.^11^ Approximately half of the PLS‐class members with E domains contain ≈136‐amino‐acid DYW domains.^12^, ^13^, ^14^ The E and DYW domains are involved in RNA editing via a reaction that deaminates specific cytidines (C) to uridines (U) in plant organelles.^15^, ^16^ Unlike PPR proteins in the PLS‐class subfamily, most P‐class PPR proteins do not possess additional domains. Nevertheless, a few P‐class subfamily members contain small MutS‐related (SMR) domains following an array of P‐class PPR motifs.^17^ These P‐class PPR proteins with SMR domains are referred to as PPR‐SMR proteins.

PPR‐SMR proteins represent a relatively small subset of PPR family members in plants.^17^ For instance, only eight PPR‐SMR proteins have been identified in Arabidopsis. These proteins contain between 4 and 11 PPR‐motif repeats; five of the eight proteins are predicted to be localized to chloroplasts, with other three being predicted to be mitochondrial. Bayesian phylogenetic analysis demonstrated that orthologs of all Arabidopsis PPR‐SMR proteins are present in the major angiosperms, including both monocots and dicots.^17^

Although few in number, PPR‐SMR proteins play essential roles in chloroplast retrograde signaling and biogenesis.^10^, ^17^, ^18^ SMR domains in other organisms possess endonucleolytic activity, suggesting that PPR‐SMR proteins could have unique functions related to the presence of this domain.^19^, ^20^, ^21^, ^22^, ^23^, ^24^ Indeed, we recently found that the PPR motif of the PPR‐SMR protein SOT1 confers RNA sequence specificity while its SMR domain confers endonucleolytic activity.^25^

The evolution, localization, and possible functions of PPR‐SMR proteins in plants have been summarized in a previous review.^17^ Here, we focus on recent progress in uncovering the roles of PPR‐SMR proteins in general and the SMR domain in particular, primarily in Arabidopsis and maize. We also discuss the potential use of SOT1 as a tool for RNA manipulation.

## 2 Roles of PPR‐SMR Proteins in Plants

PPR‐SMR protein functions have been studied mainly in Arabidopsis and maize, with only a few studies in rice. The current data on PPR‐SMR proteins come from a combination of genetic, biochemical, and physiological analyses. Thus far, there are no reports about AT2G17033, AT1G18900, AT1G74850, or their orthologs. In addition, there is only basic information on a mitochondrion‐localized PPR‐SMR protein EMB2217 that is required for embryo development and seed germination in Arabidopsis.^26^ Below, we describe recent progress in determining the functions of four chloroplast‐localized PPR‐SMR proteins (pTAC2, GUN1, SVR7/ATP4/OsPPR676, and SOT1/PPR53).

### 2.1 pTAC2

In Arabidopsis, the loss of pTAC2 results in pale yellow‐green primary leaves and seedling‐lethal phenotype. Chloroplast biogenesis and plastid‐encoded bacterial‐type RNA polymerase (PEP) dependent transcription are severely impaired in *ptac2* mutants.^27^ In rice, the loss of pTAC2 results in a more extreme phenotype compared to Arabidopsis; the rice mutant is albino.^28^ Chloroplasts in *osptac2* mutants lack thylakoids, which are replaced by empty vesicles, indicating that chloroplast biogenesis is arrested at an early stage of development in these mutants. PEP‐dependent transcription is also defective in *osptac2* mutants.^28^ These observations suggest that pTAC2 is required for PEP function in both Arabidopsis and rice. Thus, it appears that the function of pTAC2 is conserved in monocots and dicots.

### 2.2 GUN1

Chloroplast development and function relies on the coordinated expression of genes in both chloroplast and nuclear genomes by anterograde and retrograde signals. It is widely thought that retrograde signaling is of fundamental importance, in particular during chloroplast biogenesis and under various conditions where chloroplasts are stressed.^29^, ^30^, ^31^, ^32^, ^33^, ^34^, ^35^, ^36^ The first genetic screen for mutants defective in plastid‐to‐nucleus retrograde signaling resulted in the discovery of six *GENOMES UNCOUPLED* (*GUN*) loci.^37^ The *gun* mutants are characterized by their capacity to express photosynthesis‐associated nuclear genes (PhANGs) after exposure to norflurazon (NF), an inhibitor of carotenoid biosynthesis, while NF efficiently blocks expression of PhANGs in wild‐type plants. GUN2 to GUN6 are enzymes involved in tetrapyrrole biosynthesis, representing one of the retrograde signaling pathways.^38^, ^39^, ^40^, ^41^, ^42^ By contrast, GUN1 is a chloroplast‐localized PPR‐SMR protein and has not been implicated in tetrapyrrole biosynthesis.^43^ GUN1 plays an important role in multiple stress‐related retrograde signaling pathways, including those related to tetrapyrrole biosynthesis, plastid gene expression, and the redox state of the photosynthetic electron transport chain.^43^ Recently, it was clarified that GUN1 also plays a role in chloroplast development since *gun1* mutants show a hypersensitive phenotype to NF and lincomycin (Linc, a chloroplast‐specific protein synthesis inhibitor).^44^, ^45^ Thus, GUN1 plays a role in both early chloroplast development and retrograde signaling.

Interestingly, the GUN1 protein is present at very low levels and hardly detectable by proteomic approaches, while other PPR‐SMR proteins are particularly abundant compared with most PPR proteins.^17^ It was found recently that GUN1 is rapidly turned over when chloroplast biogenesis has been completed, providing a possible explanation for its low abundance. GUN1 accumulates at high levels only during very early chloroplast development and under stress conditions that involve retrograde signaling such as NF and Linc treatments,^46^ further defining the role of GUN1 in both chloroplast biogenesis and retrograde signaling. The lack of visible phenotypes of mature *gun1* mutant plants under normal growth conditions suggests that GUN1 is maintained at very low levels under unstressed conditions when its function may be not required.^43^, ^46^

Although there are numerous studies about GUN1, its exact biochemical mechanism and its precise role in retrograde signaling remain unknown.^44^ For further information about GUN1 and plastid retrograde signaling, we direct readers to recent detailed reviews.^31^, ^33^, ^35^, ^36^, ^42^

### 2.3 SVR7/ATP4/OsPPR676

SVR7 is a PPR‐SMR protein localized to chloroplasts, and its functions have been revealed by characterizing mutants. The *svr7* (*suppressor of variegation7*) mutant was initially isolated in an ethyl methanesulfonate mutagenesis screen as a suppressor of *var2* variegation.^47^ The mutation of *VAR2* (also designated *FtsH2*), encoding a chloroplast thylakoid membrane‐localized metalloprotease, alters the developmental patterns of leaves, leading to the production of variegated true leaves with obvious green and yellow (or white) sectors.^47^, ^48^ The *svr7* allele is recessive epistatic to *var2*, as the phenotype of the *svr7 var2* double mutant resembles that of the *svr7* single mutant; both display pale‐green leaves lacking white sectors.^47^, ^48^, ^49^, ^50^

**Figure 1:**
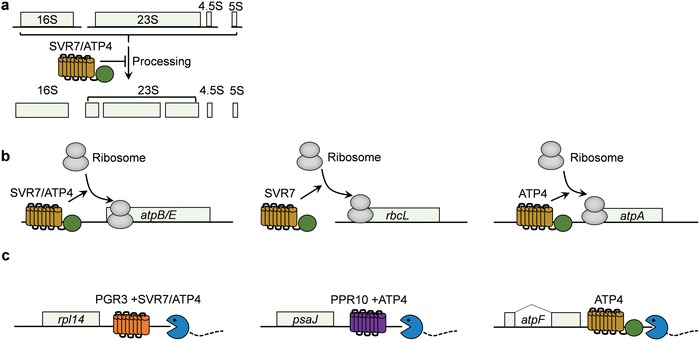
Proposed models for the possible roles of SVR7 in Arabidopsis and its maize ortholog, ATP4. a) SVR7 is directly or indirectly involved in chloroplast rRNA processing. b) SVR7 and/or ATP4 enhance translational efficiency in *atpB/E* (SVR7/ATP4), *rbcL* (SVR7), and *atpA* (ATP4). c) SVR7 and/or ATP4 enhance the stabilization of *rpl16*−*rpl14* dicistronic RNA (SVR7/ATP4, cooperating with PGR3), *psaJ* transcripts (ATP4, cooperating with PPR10), and *atpF* transcripts (ATP4) by blocking the invasion of 3′ to 5′ exonucleases. See related text for details.

Intriguingly, an unexpected functional versatility of SVR7 has been described. The loss of SVR7 results in significantly increased levels of the 23S rRNA precursor and significantly reduced levels of mature 23S rRNA. In addition, the 16S rRNA precursor and the 23S−4.5S rRNA dicistronic precursor accumulate in *svr7* mutants. These results suggest that SVR7 plays a role in chloroplast rRNA processing (**Figure** **1**a).^47^ SVR7 also increases the association of ribosomes with *atpB/E* and *rbcL* mRNAs, suggesting that it acts as a translational activator for these genes (Figure 1b).^51^ Moreover, the major dicistronic spliced *rpl16*−*rpl14* transcripts are absent in *svr7* and *pgr3* mutants, and the transcript patterns of *svr7* and *pgr3* mutants are similar, suggesting that SVR7 may cooperate closely with PGR3 to stabilize *rpl16*−*rpl14* dicistronic RNA (Figure 1c).^52^ Although the architecture of SVR7 resembles that of GUN1, *svr7* did not display the “genome uncoupled” phenotype upon treatment with NF, indicating that SVR7 is not involved in the NF‐generated retrograde signaling pathway.^51^

Most suppressors of *var2* identified to date show defects in chloroplast rRNA processing, translational machinery components, or related processes.^53^ Therefore, it is likely that the suppression of *var2* variegation caused by *SVR7* mutation is related to the modest perturbation of chloroplast rRNA processing.^47^

ATP4 is the ortholog of SVR7 in maize. The loss of SVR7 leads to the production of pale‐green seedlings. Although the *svr7* mutant is smaller than the wild type, it can complete its lifecycle.^47^ However, the loss of ATP4 results in visible chlorosis and a seedling‐lethal phenotype in maize.^54^ ATP4 plays diverse roles in post‐transcriptional control (Figure 1). Like SVR7, ATP4 promotes translational efficiency by strengthening the association of mRNA with ribosomes, specifically by binding to the 5′ ends of dicistronic *atpB/E* mRNA. ATP4 might also cooperate with PGR3 to stabilize *rpl16*−*rpl14* dicistronic RNA at its 3ʹ ends, mostly likely by blocking 3′ to 5′ degradation.^51^, ^52^, ^54^, ^55^ Maize *atp4* mutants have slightly higher levels of rRNA precursors than the wild type, which is likely a secondary effect.^54^ Unlike SVR7, ATP4 promotes the translation efficiency of *atpA*. Also, ATP4, together with PPR10, is required to stabilize the 3ʹ‐end downstream of *psaJ* from exonuclease invasion. In addition, ATP4 is required to stabilize the 3′‐end downstream of *atpF* mRNA by blocking its degradation by 3′ to 5′ exonucleases.^52^, ^54^, ^56^ Therefore, it appears that ATP4 has functionally diverged from SVR7 (Figure 1).

OsPPR676, the rice ortholog of SVR7 and ATP4, has also been characterized.^57^ The loss of OsPPR676 results in plants with slightly pale‐green leaves and significantly retarded growth. Like SVR7 and ATP4, OsPPR676 also promotes the translation of *atpB* in chloroplasts. Interestingly, however, approximately half of the pollen grains are sterile and shrunken in *osppr676* compared to the wild type. OsPPR676 interacts with Osj10gBTF3, the β‐subunit of the nascent polypeptide‐associated complex, which is crucial for pollen development.^58^ This finding suggests that OsPPR676 plays an important role in pollen development,^57^ indicating that a novel function for this protein has evolved in rice.

### 2.4 SOT1/PPR53

The roles of SOT1, a chloroplast‐localized PPR‐SMR protein, have been well examined by genetic studies. The *sot1* (*suppressor of thf1*) mutant was initially identified as a suppressor of the variegated leaf phenotype of the Arabidopsis *thf1* (*thylakoid formation 1*) mutant.^59^, ^60^, ^61^ The loss of SOT1 results in smaller plants with pale‐green cotyledons compared to wild‐type Arabidopsis. The *sot1 thf1* double mutant does not display variegated leaves with white sectors, instead having a phenotype similar to that of the *sot1* single mutant. Interestingly, the *sot1* mutant also suppresses the leaf variegation phenotype of *var2*. In addition, the *svr7* mutant recovers the variegated leaves of *thf1* and *var2* to a more normal‐like pattern resembling those of wild‐type plants.^61^ These genetic studies suggest that *sot1* and *svr7* share similar molecular mechanisms to control leaf variegation. It should be noted that the *sot1* mutant cannot rescue the expression of PhANGs in plants treated with NF, indicating that like SVR7, SOT1 is not involved in the NF‐generated retrograde signaling pathway.

The loss of SOT1 results in increased levels of the 23S−4.5S rRNA precursor and considerably reduced levels of mature 23S and 4.5S rRNA,^25^, ^61^ indicating that SOT1 is essential for the maturation of 23S and 4.5S rRNA. Since SOT1 binds to the 5′ region of the 23S−4.5S rRNA precursor via its PPR domain, Wu et al. suggested that SOT1 protects the 23S−4.5S rRNA precursor via a barrier mechanism similar to that described for some PPR proteins lacking SMR domains and thus facilitates the maturation of 23S and 4.5S rRNA.^61^ To investigate whether the PPR domain of SOT1 is fully responsible for the maturation of 23S and 4.5S rRNA, we complemented the *sot1* mutants with a construct expressing the PPR domain of SOT1 alone.^25^ The 3.2‐knt 23S−4.5S rRNA precursor was recovered in transgenic plants (*sot1‐3*/*35S:SOT1*_ppr_‐*HA*), unlike in the *sot1‐3* mutant, confirming that the PPR motifs of SOT1 indeed play a role in protecting the 23S−4.5S rRNA precursor.^61^ However, the levels of mature 23S and 4.5S rRNA in transgenic plants were only partially recovered as compared to those in wild‐type plants, indicating that the SMR domain of SOT1 plays a role in the maturation of 23S and 4.5S rRNA.

Indeed, we found that the SMR domain of SOT1 has endonuclease activity and cleaves the 23S−4.5S rRNA precursor position −38 relative to the 5′ end of mature 23S rRNA in vitro. The cleavage at the –38 site was detected in vivo, as the ≈35 nt small RNA corresponds exactly to the 5′ RNA fragment expected from cleavage at the –38 site.^61^ It should be noted that the cleavage site is ≈20 nt 3′ of the SOT1 binding site.^25^ That is a very large gap that exceeds the diameter of the SMR domain of SOT1, suggesting that some form of RNA structure is needed to bring the cleavage site within the reach of the SMR domain. The sequence around the cleavage site might be constrained to generate an RNA structure that could be cleavable by the SMR domain of SOT1. It should also be noted that a stable 3.2‐knt 23S−4.5S rRNA precursor with no cleavage at the –38 site occurs in wild‐type plants.^25^, ^61^ How does SOT1 bind to this precursor to protect it from 5′→3′ exonucleolytic degradation but without cleaving it? As discussed above, cleavage at the –38 site appears to conditional and requires the formation of some form of RNA structure around the cleavage site. It is likely that this RNA structure is not generated immediately once SOT1 binds to 5′ end of 23S−4.5S rRNA precursor, thus resulting in a stable 3.2‐knt 23S−4.5S rRNA precursor.

Mini‐ribonuclease III cleaves the 23S−4.5S precursor to simultaneously produce the mature 5′ end of 23S and the 3′ end of 4.5S.^62^ The site cleaved by the SMR domain by SOT1 is located ≈40 nucleotides upstream of the mini‐ribonuclease III cleavage site. Thus, we compared to the 5′ end of 23S rRNA and the 3′ end of 4.5S rRNA in *sot1* mutants versus mini‐ribonuclease III mutant *rnc3/4*. Mini‐ribonuclease III processing was disturbed in the *sot1* mutants.^25^ However, mini‐ribonuclease III cleavage may occur without prior cleavage at the –38 site in *sot1‐1* (a point mutation within the SMR domain of SOT1).^61^ These results suggest that endonucleolytic cleavage performed by the SMR domain of SOT1 helps facilitate the processing by mini‐ribonuclease III during the maturation of 23S and 4.5S rRNA.

**Figure 2:**
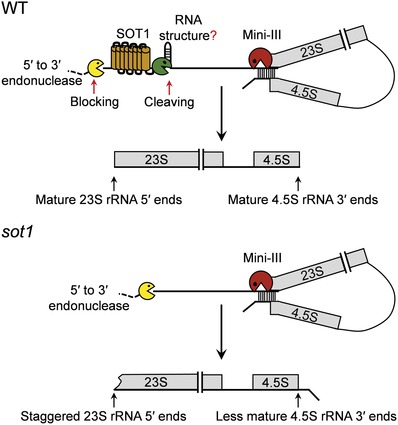
Proposed model for the role of SOT1 in chloroplast 23S−4.5S rRNA maturation in Arabidopsis. When SOT1 is present, it binds to the 5′ region of the chloroplast 23S−4.5S rRNA precursor via the PPR motifs to block 5′ to 3′ exonuclease invasion. The SMR domain of SOT1 cleaves the 23S−4.5S rRNA precursor at the −38 site. This cleavage appears to conditional and requires the formation of some form of RNA structure around the cleavage site. SOT1 helps facilitate the processing by mini‐ribonuclease III (Mini‐III) during the maturation of 23S and 4.5S rRNA. It appears that SOT1 binding is crucial to maturation of 23S and 4.5S rRNA. When SOT1 is missing, 5′ to 3′ exonuclease invades the 23S rRNA and the cleavage at the −38 site does not occur, which results in a defect in maturation of 23S and 4.5S rRNA.

Based on the current knowledge of SOT1, we propose a working model for the role of SOT1 during the maturation of 23S and 4.5S rRNA (**Figure** **2**).

PPR53, the maize ortholog of SOT1, has also been characterized.^63^ The loss of PPR53 results in a chlorotic and seedling‐lethal phenotype. The *ppr53* mutants show strong defects in the maturation of 23S and 4.5S rRNA. PPR53 binds with high affinity and specificity to the 5′ region of 23S rRNA. These findings suggest that PPR53 protects the 5′ region of 23S rRNA and promotes the maturation of 23S and 4.5S rRNA. Moreover, there was strong decrease in the expression of *ndhA* and the translational efficiency of residual *ndhA* mRNA in the mutants. However, PPR53 does not directly bind to the 5′ region of *ndhA* mRNA with high affinity. The authors conclude that PPR53 plays important roles in stabilizing RNA and enhancing translation at the *ndhA* locus, probably via the participation of other unknown factors.^63^

## 3 Roles of the SMR Domain

### 3.1 Roles of the SMR Domain in Nonplant Species

Although the SMR domain was originally described as a C‐terminal domain of the MutS2 protein in the cyanobacterium *Synechocystis*,^64^ proteins containing the SMR domain are ubiquitous in prokaryotic and eukaryotic species.^23^, ^64^ However, the biological functions of the SMR domain in nonplant species remain largely unknown. To date, studies of SMR domain‐containing proteins have mainly focused on their specific role as endonucleases. The SMR domain of human BCL‐3‐binding protein (B3BP) was first shown to have DNA endonuclease activity in 2003.^19^ The SMR domain of B3BP was also shown to have DNA‐binding activity.^21^ DNA endonuclease activity and DNA‐binding activity were also confirmed for the SMR domain of MutS2 in *Thermus thermophilus* and the “stand‐alone” SMR domain‐containing protein, YdaL, in *Escherichia coli*.^20^, ^24^ The DNA endonuclease activity of the SMR domain of MutS2 allows it to cleave branched DNA to suppress homologous DNA recombination.^20^, ^65^, ^66^ The *Leishmania donovani* RNA cycling sequence‐binding protein (LdCSBP), containing a CCCH Zn‐finger RNA‐binding domain and an SMR domain, has RNA endonuclease activity, but the full‐length protein shows only sequence‐specific RNA cleavage activity.^22^ Notably, the SMR domain of LdCSBP alone exhibits DNA and RNA endonuclease activity. Whether the SMR domain for other SMR‐containing proteins has both DNA and RNA endonuclease activity requires further study.

### 3.2 Roles of the SMR Domain in PPR‐SMR Proteins in Plants

The roles of the SMR domain in plant PPR‐SMR proteins have not yet been extensively investigated. The only known role of the SMR domain in plants was uncovered from the characterization of SOT1 in our laboratory.^25^ Like the SMR domain of LdCSBP, the SMR domain of SOT1 alone exhibits both DNA and RNA endonuclease activity. The SMR domain of SOT1 contains two conserved motifs, LDXH and TGXG.^17^ We found that the TGXG motif is critical for RNA but not DNA cleavage, suggesting that this motif is responsible for the RNA endonuclease activity of SOT1. Indeed, genetic analysis confirmed that this amino acid plays an important role in the maturation of 23S and 4.5S rRNA. By contrast, the LDXH motif is responsible for DNA endonuclease activity, and the second and fourth amino acids within the LDXH motif are critical for DNA endonuclease activity.^25^

Since the SMR domain of SOT1 has both DNA and RNA endonuclease activity, we investigated whether full‐length SOT1 exhibits both activities. Full‐length SOT1 did not cleave plasmid DNA or Arabidopsis total rRNA. However, SOT1 specifically binds to the 5′ region of the 23S−4.5S rRNA precursor, and we found that full‐length SOT1 cleaves the rRNA precursor near position −40 relative to the 5′ end of mature 23S rRNA. Thus, we conclude that full‐length SOT1 acts as a sequence‐specific RNA endonuclease in vivo, where the PPR domain confers RNA sequence specificity and the SMR domain confers RNA endonuclease activity.^25^

It is currently unknown whether the SMR domains of other PPR‐SMR proteins have DNA and/or RNA endonuclease activity. The SMR domain in plants contains two motifs, LDXH and TGXG. Based on the conservation of the LDXH and TGXG motifs (**Table** **1**), together with the biochemical characteristics of the LDXH and TGXG motifs as revealed by SOT1,^25^ we could predict whether the SMR domains of other PPR‐SMR proteins have endonuclease activity (**Table** **2**). These predictions would provide insights into the molecular mechanisms of PPR‐SMR proteins but require rigorous experimental verification.

**Table 1:** LDXH and TGXG motifs of the SMR domain in PPR‐SMR proteins in Arabidopsis and other organisms^17^

Accession number/name	LDXH motif (DNA endonuclease activity)	TGXG motif (RNA endonuclease activity)
AT5G46580/SOT1^a)^	^b)^LDVR	TGTG
AT2G31400/GUN1	LDLH	TGWG
AT1G74850/pTAC2	VDVH	SVRG
AT4G16390/SVR7	LHLK	TGHG
AT2G17033	LDLH	SGSG
AT1G79490/EMB2217	LDVR	TGPT
AT1G74750	INLH	TGWG
AT1G18900	INLH	TGWG
Hs_B3BP^c)^	LDLH	TGRG
Ld_CSBP^d)^	LDLH	TGQG

^a)^ The LDXH and TGXG motifs in the SMR domain of SOT1 confer DNA and RNA endonuclease activity, respectively^25^

^b)^ the conserved residues in LDLH and TGXG motifs are highlighted with underlines

^c)^ *Leishmania donovani* cycling sequence binding protein

^d)^ *Homo sapiens* BCL3 binding protein.

**Table 2:** Prediction of the DNA/RNA endonuclease activity of the SMR domain in various PPR‐SMR proteins in Arabidopsis

Accession number/name	DNA endonuclease activity	RNA endonuclease activity
AT5G46580/SOT1^a)^	√^b)^	√
AT2G31400/GUN1	√	√
AT1G74850/pTAC2	?^c)^	?
AT4G16390/SVR7	?	√
AT2G17033	√	?
AT1G79490/EMB2217	√	?
AT1G74750	?	√
AT1G18900	?	√

^a)^ The DNA/RNA endonuclease activity of the SMR domain has been confirmed experimentally^25^;

^b)^ ‘√' represents that the SMR domain of a PPR‐SMR protein could be predicted to possess endonuclease activity;

^c)^ ‘?' means that it is difficult to predict whether the SMR domain of a PPR‐SMR protein possess endonuclease activity.

## 4 Possible Mechanism of PPR‐SMR Proteins

During the last several years, one of the major advances in understanding the function of PPR‐SMR proteins is the elucidation of the role of the SMR domain of SOT1.^25^ The characteristics of the SMR domain of SOT1, together with genetic studies about the PPR‐SMR proteins, provide an opportunity to discuss the possible biochemical or molecular mechanism of pTAC2, GUN1, and SVR7.

### 4.1 pTAC2

pTAC2 is required for PEP function. However, the role of pTAC2 in maintaining PEP activity remains unknown. pTAC2 was originally identified as a component of plastid transcriptionally active chromosomes (pTACs) from Arabidopsis and white mustard (*Sinapis alba*).^27^ In white mustard, pTAC2 is also a key subunit in the PEP complex.^67^ In addition, the maize ortholog of pTAC2 is enriched in nucleoids.^68^, ^69^, ^70^, ^71^ Thus, it is possible that pTAC2 forms part of a transcriptionally active complex that is required for the expression of PEP‐transcribed genes.^27^ As discussed above, it is difficult to predict whether the SMR domain of pTAC2 has endonuclease activity (Table 2). Since PEP requires plastid translation for synthesis of its subunits, alternatively it is likely that pTAC2 has a role in the biogenesis of the translation machinery, in particular in rRNA processing.

### 4.2 GUN1

Due to the central role of GUN1 in retrograde signaling, many efforts have been invested in elucidating its molecular mechanism.^46^, ^72^, ^73^, ^74^, ^75^ However, its biochemical mechanism and role in retrograde signaling remain enigmatic. A major problem is that *gun1* mutants show no visible phenotype that distinguishes them from wild‐type plants under normal growth conditions and thus are difficult to study. Furthermore, it has proven difficult to obtain an antibody that works for native GUN1, despite multiple attempts.^44^ GUN1 is also present very low abundance due to its rapid degradation under normal growth conditions.^46^ These problems have hampered the identification of GUN1's targets and the discovery of its precise molecular mechanism.^43^, ^74^

To gain insight into the molecular mechanism of GUN1, proteomic approach has been employed to identify its interacting proteins using overexpressed GUN1‐GFP transgenic plants. The large number of GUN1‐interacting proteins identified this way represents a wide range of functions involved in ribosome biogenesis, tetrapyrrole biosynthesis, protein import, and protein homeostasis.^74^ However, the overexpression of GUN1‐GFP is prone to lead to the identification of false interacting proteins.^76^ Given the wide diversity of proteins identified, the proteomic attempts have so far provided few clues about the true functions of GUN1.^36^

What might be the precise role of GUN1 in chloroplast biogenesis and retrograde signaling? The impaired repression of PhANGs in *gun1* mutants occurs when the seedlings are treated with plastid translational inhibitors such as Linc or rifampicin, suggesting that GUN1 is required for retrograde signaling via plastid gene expression.^33^, ^43^, ^77^, ^78^ In general, PPR proteins bind RNA and are involved in RNA metabolism and thus in plastid gene expression.^5^, ^7^ Therefore, one obvious target for GUN1 might be the plastid‐transcribed RNA. Recently, genetic studies have shown that both the PPR motifs and the SMR domain are required for GUN1 function in retrograde signaling.^46^ The point mutation within the SMR domain in *gun1‐11* results in a typical *gun* phenotype, which also strongly implies that the SMR domain of GUN1 is involved in retrograde signaling.^43^ Moreover, the GUN1 SMR domain is predicted to possess RNA endonuclease activity (Table 2). The biochemical characteristics of the PPR motifs and the SMR domain of GUN1 indicate that GUN1 could be a sequence‐specific RNA endonuclease. Thus, we tentatively speculate that GUN1 has a role in plastid RNA metabolism. Future endeavors should aim to identify RNA targets of GUN1.

### 4.3 SVR7

SVR7 is suggested to play a role in chloroplast 23S rRNA processing.^47^ There are two known cleavage sites inside 23S rRNA precursor to generate mature 23S rRNA transcripts (0.5, 1.1, and 1.3‐knt 23S rRNA transcripts). The first cleavage site generates mature 0.5‐knt transcripts. The second cleavage site produces 1.3 and 1.1‐knt mature transcripts, as well as 1.8‐kb 23S rRNA precursor. Compared with those in wild‐type plants, the levels of the 2.9 and 2.4‐knt 23S rRNA precursors are significantly increased while those of the 1.8, 1.3, and 1.1‐knt 23S rRNA transcripts are significantly decreased in the *svr7* mutant. Interestingly, there is no obvious change in the level of the 0.5‐knt 23S rRNA transcripts in the *svr7* mutant.^47^ This suggests that there is a processing defect in the second cleavage site. Given that the SVR7 SMR domain may confer RNA endonuclease activity (Table 2), we deduce that SVR7 is involved in the processing of mature 1.3 and 1.1‐kb 23S rRNAs by acting as an RNA endonuclease.

## 5 SOT1 Represents a Promising Tool for RNA Manipulation

DNA restriction enzymes were discovered ≈40 years ago and were subsequently developed as a set of tools for DNA manipulation. Considering the critical roles of RNAs, tools for RNA manipulation would be an attractive target for development. RNA manipulation has several advantages compared to DNA manipulation: (1) it does not result in permanent changes to the genome^79^; (2) it can be used as an RNA silencing tool to complement RNAi, which is sometimes ineffective in certain organisms^80^; (3) it can be used as an RNA silencing tool for organelle genes, since RNAi machinery is not present in organelles such as chloroplasts and mitochondria and CRISPR/Cas9 is thought to be ineffective for these organelles due to the difficultly in importing guide RNAs into organelles.^80^, ^81^ Thus, huge efforts have focused on identifying sequence‐specific RNA endonucleases.

To date, many RNA binding proteins have been shown to recognize RNA sequences and/or structures via their modular structures, such as zinc finger, K homology, and Pumilio/FBF homology (PUF) proteins.^82^ Among these RNA binding proteins, the classic PUF protein contains eight PUF repeats that recognize eight consecutive RNA nucleotides using an elegant one‐repeat:one‐nucleotide binding mode.^83^ Moreover, the RNA specificity of the PUF domain has been well documented.^84^, ^85^, ^86^ Thus, PUF proteins represent candidates for engineering RNA endonucleases that recognize RNA in a highly sequence‐specific manner. Indeed, an engineered PUF motif with the ability to perform programmable RNA recognition has been developed. More importantly, the PUF domain fused with the PIN domain of SMG6, a general RNA cleavage domain, has been successfully engineered as an artificial sequence‐specific RNA endonuclease that can specifically recognize and cleave its RNA target.^80^

Like PUF proteins, canonical PPR proteins also possess a modular configuration and can specifically recognize RNAs in an intrinsic sequence‐specific manner.^87^, ^88^ The 2nd, 5th, and 35th residues at each PPR motif are considered to be RNA selection “codes.”^87^, ^88^, ^89^ Based on these codes, two groups have made advances in artificially engineering PPR proteins that are optimized to specifically recognize their predicted RNA targets.^90^, ^91^, ^92^ Recently, it has been demonstrated that the modified RPF2 (a mitochondrial PPR protein) binds a new target located within the coding sequence of *nad6* and specifically induces cleavage of *nad6* RNA, almost eliminating expression of the Nad6 protein in Arabidopsis.^93^ This represents the first example of a targeted block in expression of a specific mitochondrial transcript by a custom‐designed RNA‐binding protein. Therefore, it is likely that PPR proteins could be engineered into custom RNA‐binding proteins with high specificity. On the other hand, a synthetically designed ribozyme led to directed knockdown of *matR* expression, suggesting that ribozymes could also be used as a tool for RNA manipulation.^94^, ^95^

It is currently unknown whether a PPR protein when fused with an RNA endonuclease domain can be engineered as an artificial sequence‐specific RNA endonuclease that specifically recognizes and cleaves its RNA target. As discussed above, PPR proteins have the capacity for sequence‐specific RNA binding. If the SMR domain of PPR‐SMR proteins is confirmed to have nuclease activity, these proteins would represent natural sequence‐specific RNA endonucleases. Indeed, we recently confirmed that the SMR domain of the PPR‐SMR protein SOT1 has RNA endonuclease activity.^25^ We also demonstrated that SOT1 serves as a sequence‐specific RNA endonuclease. Moreover, we successfully engineered SOT1 to recognize and cleave RNA with customizable sequence specificity. Therefore, SOT1 might serve as an exciting tool for RNA manipulation.^25^

Chloroplast transformation is especially easy in *Chlamydomonas* and is feasible in other plants with practical applications. To date, chloroplast transformation has been achieved in some plant species including tobacco, Arabidopsis, solanaceous species, lettuce, and poplar.^96^, ^97^, ^98^, ^99^, ^100^, ^101^ However, extending the species range of the chloroplast transformation technology has proven extremely difficult, in particular for major agricultural crops.^101^, ^102^ Mitochondrial transformation has been realized only in the budding yeast *Saccharomyces cerevisiae* and *Chlamydomonas reinhardtii* and is very difficult in most organisms (including plants) since there is lack of viable transformation approaches.^93^, ^103^ In addition, the function of many open reading frames in chloroplast and mitochondrial genomes in major agricultural crops, such as rice and maize, remains unknown. Although SOT1 is a chloroplast‐localized protein, it can also be targeted to mitochondria by replacing its signal peptide with a mitochondrial signal peptide. Thus, SOT1 represents a potential tool for use in gene knockdown experiments for both chloroplasts and mitochondria. Crops are often attacked by various RNA viruses. In an applied context, it is possible that SOT1 could be used as an RNA manipulation tool to help defend against RNA viruses in crops.

To better use SOT1 as an RNA manipulation tool, we need engineer an analogous, artificial PPR‐SMR system that recognizes targeted RNA with high specificity and cleaves it with high efficiency. Based on modular design principles, an artificial PPR‐SMR system should be possible to construct by combining the PPR domain and the SMR domain.

Indeed, an artificial PPR domain that can effectively recognize targeted RNA has already been successfully designed.^90^, ^91^, ^92^ 35‐amino‐acid PPR repeat scaffolds could be constructed that can specifically bind to RNA bases A, U, C, and G. Recently, numerous PPR codes have been deciphered recently, which facilitates the design of PPR repeat scaffolds to recognize degenerate bases.^104^ Also, an effective, convenient method for custom assembly of designer PPRs has been provided.^104^ Thus, constructing an artificial PPR domain that can effectively recognize targeted RNA should be realized easily and conveniently.

Identifying an SMR domain with high RNA endonuclease activity is also critical for designing an artificial PPR‐SMR system. Several questions remain to be addressed. First, we need to identify an SMR domain with high RNA endonuclease activity by screening the SMR domains in various organisms. Second, elucidating the detailed catalytic mechanism of the SMR domain will be invaluable for optimizing the RNA endonuclease activity of the SMR domain. Finally, we need to investigate whether the SMR domain displays bias for its substrate elements, such as RNA structure and base preference. If so, these elements must be identified.

## 6 Conclusions and Outlook

PPR‐SMR proteins comprise only a small subset of the PPR family. Increasing evidence indicates that PPR‐SMR proteins have crucial and diverse functions, primarily involving organelle gene expression. There is currently a lack of information about the functions of three of the eight PPR‐SMR proteins in Arabidopsis (AT2G17033, AT1G18900, and AT1G74850). Although functions of several PPR‐SMR proteins, such as ATP4 and SOT1, have been characterized, we still lack information about the specific mechanisms of PPR‐SMR proteins. Critical future directions for the study of PPR‐SMRs in plant organelles include efforts to (1) investigate how PPR‐SMRs are involved in organellar gene expression and their underlying effects on transcription, RNA metabolism, and translation; (2) verify the endonuclease activity of the SMR domain and reveal its catalytic mechanism; and (3) develop SOT1 as a tool for RNA manipulation in the application of organellar biology and RNA viral defense in crops.

## Conflict of Interest

The authors declare no conflict of interest.
